# The Role of PIWI‐Interacting RNA in Urologic Carcinoma and Its Clinical Implications

**DOI:** 10.1002/cnr2.70272

**Published:** 2025-07-30

**Authors:** Jiajia Cai, Shirui Jiang, Dongdong Lu, Ghulam Murtaza, Mingbang Wang, Yuqing Li, Da Shi, Yingrui Li, Hu Fang, Song Wu

**Affiliations:** ^1^ Department of Experimental Research South China Hospital, Medical School, Shenzhen University Shenzhen China; ^2^ Department of Nutritional Physiology Institute of Nutritional Sciences, Friedrich Schiller University Jena Jena Germany; ^3^ Interdisciplinary Innovation Center, South China Hospital, Medical School, Shenzhen University Shenzhen China; ^4^ Institute of Biomedical Data, South China Hospital, Medical School, Shenzhen University Shenzhen China

**Keywords:** clinical implication, PIWI‐interacting RNA, urologic carcinoma

## Abstract

Piwi‐interacting RNA (piRNA) represents a class of small non‐coding RNA molecules, typically ranging in length from 18 to 35 nucleotides. These molecules are critically involved in the preservation of genomic integrity and the regulation of protein translation processes. Recently, emerging scholarly research indicates that piRNA exhibits tissue‐specific expression profiles within a variety of human malignancies, where they intricately regulate key signaling pathways at both the transcriptional and post‐transcriptional levels. This review systematically underscores current investigations pertaining to piRNA in urologic carcinoma (UC), elucidating the proposed regulatory mechanisms encompassing N6‐methyladenosine (m6A) modification and the silencing of transposable elements. Furthermore, we discuss the detection technology and the application of piRNA in the fields of clinical diagnosis.

## 1 Introduction

**FIGURE 1:**
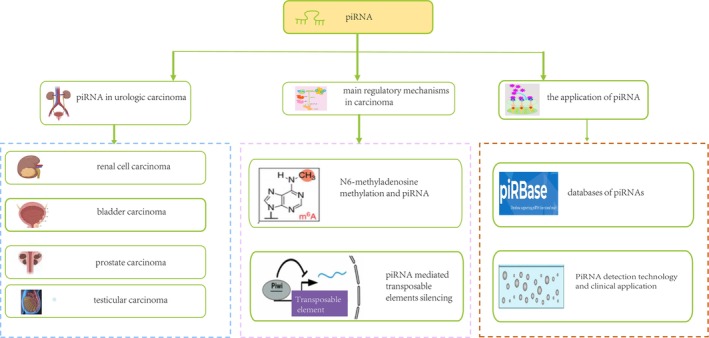
The workflow of this article.

PIWI‐interacting RNA (piRNA) represents a novel class of genetic regulatory small noncoding RNAs, earning recognition as one of the pivotal scientific breakthroughs in 2006 [1, 2, 3, 4]. Exhibiting a typical length of 18–35 nucleotides [5], piRNAs were first recognized in reproductive cells, and they are essential for the sophisticated processes involved in controlling gene expression [6, 7, 8, 9]. Unlike microRNA (miRNA) and small interfering RNAs(siRNA), which predominantly originate from double‐stranded RNA precursors, piRNAs are derived from extended single‐stranded RNA precursors [8, 9, 10]. Compared to germline cells, emerging research has suggested that piRNAs may play additional roles in somatic cells, albeit exhibiting lower expression levels [11, 12, 13, 14]. PiRNAs exert their regulatory functions in animal cells by forming complexes with PIWI analogs, specifically PIWIL proteins belonging to the Argonaute gene/protein family [15, 16]. The complex interaction between PIWIL and piRNA assumes a pivotal role in male germline development [17, 18, 19, 20]. Moreover, common piRNAs may be instrumental in propelling the proliferation of cancer cells, while specific piRNAs likely contribute to distinct cancer biology [21, 22, 23, 24, 25]. The recent research has significant interest in the examination of piRNA expression and function across various cancer types, including urologic carcinoma (UC) [26, 27, 28, 29]. Herein, we first summarize the dysregulation of piRNA in UC, and then discuss the main regulatory mechanisms, the database as well as the detection technology of piRNA (Figure 1).

### 1.1 Current Studies of piRNA in Urologic Carcinoma

#### 1.1.1 Dysregulation of PIWIL Proteins and piRNA in Renal Cell Carcinoma

Several piRNAs and PIWIL proteins were investigated in renal cell carcinoma (RCC) as well. Table 1 provides a detailed synopsis of the role of piRNA and PIWIL proteins in various contexts of RCC. The PIWIL4 gene exhibited significantly elevated expression levels in clear cell renal cell carcinoma (ccRCC) tissues [30]. The expression of PIWIL proteins is not only associated with tumors, but also correlated with the classification of those tumors. In another study, the expression of PIWIL1 contributed to a more sophisticated classification of RCC patients, particularly those exhibiting elevated Fuhrman grade, advanced tumor stage, and distant metastasis [26]. In a separate study about the outcome of patients, the results show that the expression levels of PIWIL1, PIWIL2, and PIWIL4 were positively associated with advanced clinical staging, and low expression of them reduced overall survival in ccRCC [31]. However, no difference is observed in terms of PIWIL2 and PIWIL4 expression, which are contradictory to previous research based on a cohort of 73 paired samples. This discrepancy may account for the limited sample size. Collectively, these studies revealed that PIWIL proteins correlated with the clinical stages and survival of RCC.

**TABLE 1:** Dysregulation of piRNA and PIWI proteins in urologic carcinoma.

Cancer type	piRNA/protein	Group number	Value	Test method	References
RCC	PIWIL1	One independent cohorts of 265 RCC patients and another of 345 RCC patients	PIWIL1 facilitated a refined stratification of RCC patients, particularly those exhibiting elevated Fuhrman grade, advanced tumor stage, or distant metastasis	IHC	[26]
	PIWIL1, PIWIL2, PIWIL3 and PIWIL4	57 ccRCC tissues and 38 tissues of adjacent non‐tumor renal parenchyma	PIWIL1, PIWIL2, and PIWIL4 were significantly associated with worse overall survival in RCC patients	qRT‐PCR	[31]
	piR‐823	153 tumor tissue, 121 adjacent renal parenchyma, 178 blood serum and 20 urine of patients undergoing nephrectomy for renal cell carcinoma and 102 in blood serum and 15 urines of matched healthy controls	piR‐823 down expressed significantly in tumor tissues with better disease‐free survival, increased expression in serum and urine compared to healthy subjects with advanced clinical stages	qRT‐PCR	[32]
	piR‐32 051, piR‐39 894 and piR‐43 607	24 frozen benign kidney and ccRCC specimens	piR‐32 051, piR‐39 894 and piR‐43 607 were increased and associated with ccRCC metastasis, late clinical stage and poor cancer‐specific survival	qRT‐PCR	[34]
	piR‐30 924, piR‐57 125, and piR‐38 756	76 non‐metastatic and 30 metastatic ccRCC tissue	The higher expression of piR‐30 924 and piR‐38 756 in metastatic primary tumors were significantly associated with tumor recurrence and overall survival, on the contrary, piR‐57 125 expressed lower	piRNA microarray	[35]
	piR‐1742	Tumor tissues and paired normal tissues in RCC	piR‐1742 expressed increasingly in RCC tumors tissues compared to normal tissues associated with a poor prognosis	piRNA microarray expression	[36]
	piR‐31 115	Six ccRCC tissues and matched adjacent normal tissues	piR‐31 115 was the most upregulated piRNA in ccRCC tissues compared with matched adjacent normal tissues	Deep sequencing	[37]
	piR‐57 125		piR‐57 125 restrains ccRCC metastasis by directly targeting CCL3 and inhibiting the AKT/ERK pathway	piRNA‐sequencing combined with TCGA data	[38]
	Piwi‐like 2	202 BC patients treated with cystectomy and adjuvant chemotherapy	Absent nuclear Piwil 2 immunoreactivity was significantly associated with poor disease‐specific and tumor progression of those patients	IHC	[41]
BC	Piwi‐like 1 and Piwi‐like 2	95 muscle invasive BC samples	Positivity associated with an increased risk of disease‐specific survival	IHC	[42]
	DQ594040	Three BC tissues and the adjacent normal tissues	Promoted bladder cancer cell apoptosis, colony formation, and proliferation by up‐regulating TNFSF	Microarray	[24]
PC	piR‐001773 and piR‐ 017184	24 pairs of tumor tissues and their adjacent normal tissues	The downregulation of piR‐001773 and piR‐017184 markedly inhibited tumor growth	qRT‐PCR	[45]
	piR‐19 004 and piR‐2878, piR‐ 19 166	42 pairs of PC specimens and matched normal prostate tissues	piR‐19 004 and piR‐2878 were up‐regulated, while piR‐19 166 was down‐regulated in PC	qRT‐PCR	[46]
	piR‐31 470	24 pairs of tumor tissues and their adjacent normal tissues	Down regulated the transcription of GSTP1 and increased vulnerability to oxidative stress and DNA damage in human prostate epithelial RWPE1 cells	qRT‐PCR	[47]
	piR‐651 and piR‐823	PC cell lines (LNCaP and PC‐3)	The level of piR‐651 and piR‐823 might be influenced by hormone treatment	qRT‐PCR	[48]
TC	PIWIL1, PIWIL2, and PIWIL4	17 Primary TC tissues and 19 non‐TC tissues	Hypermethylation‐associated of promoter CpG island silencing the expression of PIWIL1, PIWIL2, and PIWIL4	qRT‐PCR	[49]
	PIWIL2	Cell lines	Inhibition of apoptosis and the promotion of proliferation	qRT‐PCR	[50]
	PiR‐36 249	Rat	Downregulated in testicular cancer tissues compared to tumor‐adjacent tissues	qRT‐PCR	[25]

Abbreviations: BC, bladder carcinoma; ccRCC, clear cell renal carcinoma; IHC, immunohistochemistry; PC, prostate carcinoma; PIWIL1, piwi‐like RNA‐mediated gene silencing 1; PIWIL2, piwi‐like RNA‐mediated gene silencing 2; PIWIL3, piwi‐like RNA‐mediated gene silencing 3; PIWIL4, piwi‐like RNA‐mediated gene silencing 4; qRT‐PCR, quantitative reverse transcription polymerase chain reaction; RCC, renal cell carcinoma; TC, testicular carcinoma.

Furthermore, increasing studies have proposed that piRNA can function as epigenetic effectors in RCC. The inaugural exploration of the prognostic implications of piRNAs were conducted in ccRCC patient. Some low expression of piRNA were a favorable progression‐free factors. For instance, a substantial decrease in the expression of piR‐823 within cancer tissues has been linked to enhanced disease‐free survival rates [32]. Additionally, lower expression of both piR‐34536 and piR‐51810 were a more unfavorable progression‐free factors [33]. Similarly, the heightened expression levels of piR‐32051, piR‐39894, and piR‐43607 have been identified as being significantly correlated with the metastasis of ccRCC, as well as with advanced clinical staging and poor cancer‐specific survival [34]. The metastasized tumors, showing higher expression levels of piR‐30924 and piR‐38756, are significantly associated with an increased incidence of tumor relapse and poorer survival outcomes [35]. Additionally, some potential of these piRNA were underscored as prognostic biomarkers when combined with clinicopathological factors in primary non‐metastatic and metastatic ccRCC tissues [35], such as piR‐30924 and piR‐57125, both of them can be as potential independent predictive molecules.

**FIGURE 2:**
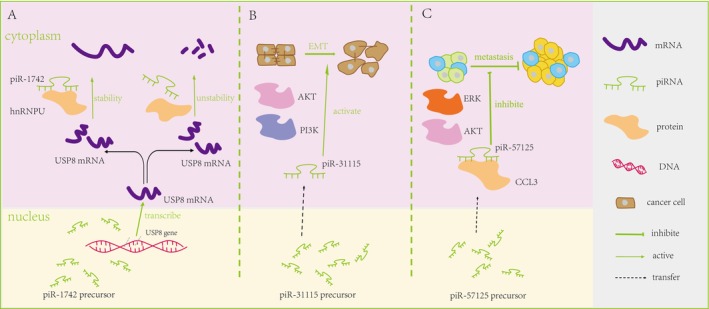
Detailed regulatory mechanism in Renal Cell Carcinoma. (A) Regulatory Interaction of piR‐1742 with USP8 mRNA Stability via hnRNPU Binding [36]. (B) piR‐31 115 activates epithelial‐mesenchymal transition (EMT) via the PI3K/AKT signaling pathway [37]. (C) piR‐57 125 restrains clear cell renal cell carcinoma metastasis by directly targeting CC chemokine 3 (CCL3) and inhibiting the Serine/Threonine Protein Kinase B (AKT)/Extracellular signal‐regulated kinase (ERK) pathway [38].

Recently, the mechanism of piRNA in RCC initiation has been elucidated. For example, there was a notable increase in piR‐1742 expression in neoplastic tissues as opposed to their normal counterparts, with higher expression levels being significantly correlated with worse clinical outcomes for patients [36]. Furthermore, studies utilizing RCC xenografts and organoids have indicated that piR‐1742 can modulate the mRNA stability of USP8 by specifically binding to hnRNPU [36] (Figure 2A). In a parallel investigation, piR‐31 115 has been identified as the most upregulated piRNA molecule, and silencing of piR‐31 115 demonstrated inhibitory effects on ccRCC cell proliferation, motility, and invasiveness [37]. Investigations into the underlying mechanisms have revealed that piR‐31 115 might facilitate the process of epithelial‐mesenchymal transition (EMT) by engaging the PI3K/AKT signaling pathway [37] (Figure 2B). However, a decreased expression of piR‐57 125 in ccRCC tissues has been observed, which is linked to the suppression of ccRCC metastasis through the direct targeting of CC chemokine 3 (CCL3) and the inhibition of the AKT/ERK pathway [38] (Figure 2C).

Moreover, in a study conducted by Martinez and colleagues, variations were identified in the homogeneous piRNA signatures that are specific to different types of cancer among patient cohorts [39]. Uniform piRNA signatures were identified in RCC patients, a cancer type marked by a consistent upregulation of hypoxia‐associated signaling pathways, which are often triggered by mutations or the absence of the von Hippel–Lindau tumor suppressor. Collectively, the research has exposed the irregularities in piRNA expression within RCC, suggesting a new pathway that may be pivotal in the genesis and advancement of tumors. In summary, the piRNA pathway genes hold promise for acting as indicators for diagnostic and prognostic assessment in ccRCC.

#### 1.1.2 Dysregulation of Piwi‐Like Proteins and piRNA in Bladder Carcinoma

Efforts are continuously being made to seek a more cost‐effective and less invasive alternative to the current BC diagnostic procedures due to their high costs and invasive nature [40]. Altered expression patterns of PIWIL proteins and piRNA between BC tissue and adjacent normal bladder tissue suggest potential involvement in the pathogenesis or progression of BC [29, 41]. While the presence of Piwi gene expression has been identified within cancer cells, a comprehensive understanding of its functional attributes and interactions with piRNA remains limited, necessitating further in‐depth investigation. Notably, a study has identified a substantial link between diminished Piwi‐like 2 expression and negative patient outcomes, including poor disease‐specific survival and tumor progression in this cohort of patients [41] (Table 1). In 2018, Eckstein et al. conducted an analysis of Piwi‐like 1 and Piwi‐like 2 expression using immunohistochemistry (IHC) in a cohort comprising 95 muscle invasive bladder carcinoma (MIBC) samples [42]. Their investigation considered Piwi‐like proteins as independent prognostic factors, with positive expression markedly linked to a heightened probability of disease‐specific mortality.

Examining piRNA obtained from three BC tissues and their corresponding normal tissues focusing on the sequences within the 3′ untranslated region (3′UTR), Chu et al. uncovered a significant number of piRNAs that were either up‐regulated or down‐regulated in BC tissues, with 106 piRNAs showing increased expression and 91 exhibiting decreased expression [24]. Functional analyses revealed that piRNA DQ594040 exerted regulatory effects on BC cell apoptosis, colony formation, and proliferation by up‐regulating TNFSF4 [24]. The outcomes were corroborated by employing the technique of quantitative reverse transcription polymerase chain reaction(qRT‐PCR) [24] (Table 1). Tumor‐specific piRNA and Piwi‐like proteins, elucidated in those studies, emerge as promising candidates for prospective biomarkers, presenting pertinent diagnostic and prognostic data and emerging as viable options for therapeutic intervention. Although still in the early stage, exploring the feasibility of targeting piRNA or the associated pathways presents a promising therapeutic strategy for BC.

#### 1.1.3 Dysregulation of Piwi‐Like Proteins and piRNA in Prostate Carcinoma

**FIGURE 3:**
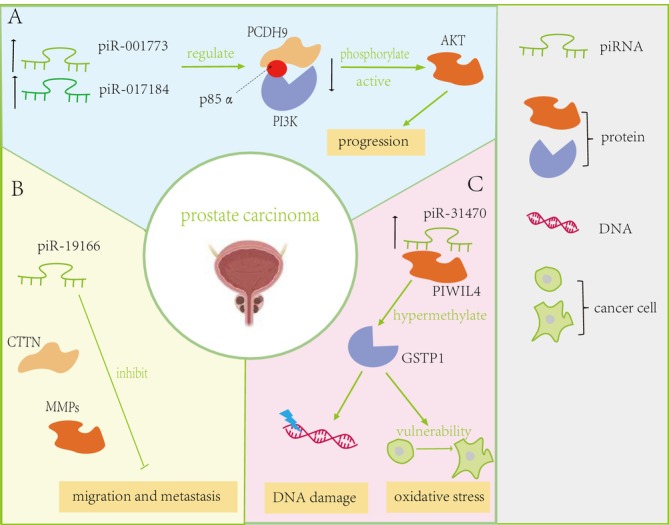
Detailed regulatory mechanism in Prostate Carcinoma. (A) piR‐001773 and piR‐017184 exert posttranscriptional downregulation on protocadherin 9 (PCDH9). This regulatory process involves the binding of these small RNAs to p85α, the regulatory subunit of phosphoinositide 3‐kinase (PI3K), culminating in heightened serine/threonine protein kinase B (AKT) phosphorylation and activation [45]. (B) piR‐19 166 was identified as a regulator of the cortactin (CTTN)/matrix metalloproteinases (MMPs) pathway, exerting inhibitory effects on the migration and metastasis of prostate carcinoma cells [46]. (C) Heightened expression of piR‐31 470, which combines with PIWIL4, maintained the hypermethylation and inactivation of glutathione S‐transferase pi‐1 (GSTP1). The overexpression of piR‐31 470 decreases the transcription of GSTP1 and increases vulnerability to oxidative stress and DNA damage in prostate carcinoma [47].

The most common UC in men is prostate cancer (PC). Combined with non‐invasive biomarkers, it can achieve high accuracy. Due to limitations in detection technology, the traditional biomarkers are mRNA, such as SelectMDx, a diagnostic method that tries to measure mRNA levels of DLX1 and HOXC6 genes. However, those diagnostic methods did not consider the diagnostic value of piRNA in PC. Extensive investigations into the role of PIWIL proteins in PC have unveiled their significance in predicting clinical outcomes. Analysis of PIWIL2 expression in serum indicated no significant difference between volunteers and PC patients, yet it exhibited relevance to the clinical outcome of PC [43]. Although unsuitable as a diagnostic indicator, elevated PIWIL2 expression holds promise as a beneficial prognostic indicator [43]. Further studies demonstrated associations between PIWIL2 expression, Gleason score, and clinical Tumor‐Node‐Metastasis (TNM) stage, with knockdown experiments revealing diminished invasive and migratory abilities in PC cells [44]. Collectively, these findings emphasize the contributory role of dysregulated PIWI proteins in PC prognosis. The emerging studies of PIWI protein in PC offer novel insights regarding the relevance of piRNA in cancer. Scientific inquiries have identified piR‐001773 and piR‐017184 as being elevated in prostate cancer specimens, and these molecules are believed to regulate the expression levels of protocadherin 9 (PCDH9) in a posttranscriptional manner, thereby influencing disease pathology [45]. This control mechanism involves the engagement with p85α, the regulatory component of the phosphoinositide 3‐kinase (PI3K) complex, culminating in heightened AKT phosphorylation and activation, facilitating prostate cancer progression [45] (Figure 3A). Conversely, the expression levels of piR‐19 004 and piR‐2878 were notably increased, in contrast to piR‐19 166, which showed a significant decrease, with piR‐19 166 identified as a regulator of the cortactin (CTTN)/matrix metalloproteinases (MMPs) pathway, inhibiting migration and metastasis of PC cellss [46] (Figure 3B). Additionally, Zhang et al. [47] have elucidated that the elevated levels of piR‐31 470 in PC cells interact with PIWIL4, forming a complex that sustains the hypermethylation and subsequent silencing of glutathione S‐transferase pi‐1 (GSTP1), thereby sensitizing these cells to oxidative stress and DNA damage [47] (Figure 3C). Collectively, these findings emphasize the pivotal impact of aberrant piRNA regulation in the advancement of PC.

Focusing on elucidating the roles and clinical potential prognostic significance of piRNA in PC. Zhao et al. [28] identified hsa_pir_000627, hsa_pir_005553 and hsa_pir_019346 as significantly correlated with biochemical recurrence of PC, marking them as novel prognostic markers [28]. Moreover, variations in piRNA expression levels between prostate cancers of Gleason score indicate that these molecules could serve as discriminating markers for the stratification of disease severity. Additionally, by exploring the impact of hormone treatment on piRNA expression in PC, elevated expressions of piR‐651 and piR‐823 post‐treatment was found and suggesting their potential responsiveness to hormonal influences [48]. In aggregate, the research indicates that piRNAs have the potential to be employed as prognostic biomarkers for PC. Moreover, these discoveries may open avenues for novel therapeutic strategies involving the targeted modulation of piRNA for epigenetic treatment of prostate carcinoma.

#### 1.1.4 Dysregulation of PIWI Protein and piRNA in Testicular Carcinoma

**FIGURE 4:**
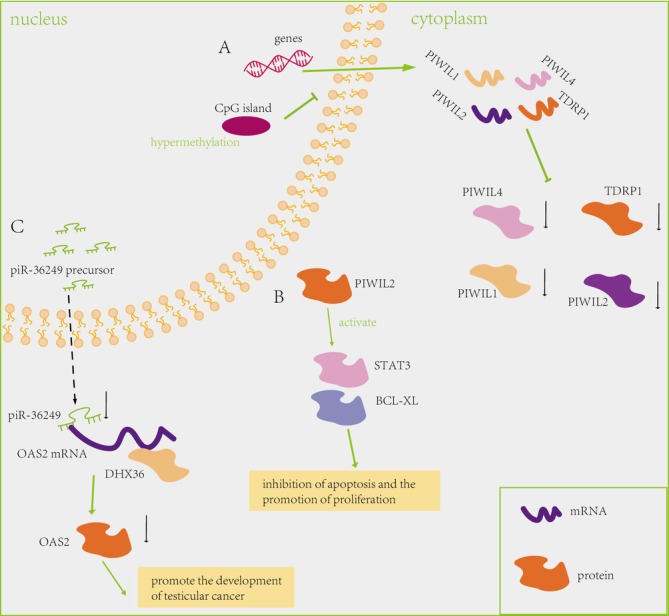
Detailed regulatory mechanism of Testicular Carcinoma. (A) Hypermethylation‐associated promoter CpG island silencing the expression of PIWIL1, PIWIL2, and PIWIL4 in testicular carcinoma [49]. (B) Widespread expression of PIWIL2 in tumors posits its role as an oncogene through the inhibition of apoptosis and the promotion of proliferation via the Signal Transducer and Activator of Transcription 3 (STAT3)/BCL‐XL signaling pathway [50]. (C) piR‐36 249 demonstrates binding affinity to the 3′ untranslated region (UTR) of the 2′‐5′‐oligoadenylate synthetase 2 (OAS2) messenger RNA (mRNA), while concurrently, DHX36 exhibits a similar capability to bind to the OAS2 mRNA. The collaborative downregulation of piR‐36 249 and DHX36 collectively contributes to the facilitation of the malignant phenotype in testicular cancer cells, achieved through the concerted downregulation of OAS2 protein levels [25].

Recent Insights Underscore the Indispensable Role of piRNAs in Testicular Carcinoma (TC). Hypermethylation‐associated promoter CpG island silencing leads to reduced levels of expression for PIWIL1, PIWIL2, and PIWIL4 in TC [49] (Figure 4A). A separate study has highlighted the ubiquity of PIWIL2 in tumorigenesis, suggesting that its role in oncogenesis is mediated by the inhibition of apoptosis and the stimulation of cell proliferation through the Stat3/Bcl‐XL signaling pathway (Figure 4B) [50]. Research has disclosed that piR‐36249 exhibits a marked reduction in expression within TC tissues as opposed to the adjacent non‐cancerous tissues, suggesting its potential role in the disease's pathology [25]. Specifically, piR‐36249 and DHX36 have been identified to interact with the 3′ untranslated region (UTR) of the 2′‐5′‐oligoadenylate synthetase 2 (OAS2) mRNA, contributing to the regulation of OAS2 expression at the post‐transcriptional level [25]. The collaborative downregulation ofpiR‐36249 and DHX36 collectively contributes to the facilitation of the malignant phenotype in TC cells, achieved through the concerted downregulation of OAS2 protein levels (Figure 4C). As the roles of piRNAs in TC continue to unfold, there is a burgeoning interest in their potential as therapeutic targets. Strategies involving the modulation of piRNA expression or activity are under consideration. However, translating these ongoing studies and potential therapies into clinical applications is expected to require several years of further research and validation.

### 1.2 The Main Regulatory Mechanisms in Carcinoma

#### 1.2.1 Crosstalk Between N6‐Methyladenosine Methylation and piRNA

In general, N6‐methyladenosine (m6A) modification and piRNA can induce oncogenesis by forming intricate networks [51, 52]. Aberrant expression of piRNA may result in epigenetic dysregulation by modulating m6A methylation and chromatin modification [52, 53, 54, 55]. Elaborating the crosstalk between major methylation modifications and piRNAs holds the potential to advance our understanding of cancer oncogenesis [54, 55]. For example, elevated levels of piRNA‐14633 have been shown to enhance cervical cancer cell viability, stimulate their proliferation, and augment both migration and invasive capabilities. Besides, piRNA‐14633 influences the malignancy of cervical cancer cells through the enhancement of m6A RNA methylation and the preservation of methyltransferase like 14 (METTL14) mRNA stability. This indicates that elevated levels of piRNA‐14633 may drive the growth, mobility, and invasiveness of cervical cancer cells through modulation of the METTL14/CYP1B1 signaling pathway [54]. Similarly, Han et al. [55] have discovered that the piRNA‐30 473/WTAP/HK2 axis is instrumental in the development of diffuse large B‐cell lymphoma by modulating m6A RNA methylation. The prognostic significance of m6A regulatory elements piRNA‐30 473 and WTAP is evident in diffuse large B‐cell lymphoma patients, underscoring their role in forecasting survival rates. In detail, WTAP elevates the expression of hexokinase 2 (HK2), a critical target gene, thereby upregulating the expression level of piRNA‐30 473 to advance large B‐cell lymphoma by boosting HK2 m6A methylation levels [55].

Furthermore, in a study conducted by Liu et al., it was found that the levels of piRNA‐36 741 and the methyltransferase like 3 (METTL3) increased during the osteogenic differentiation of bone marrow mesenchymal stem cells (BMSCs). Mechanistically, the increased expression of piRNA‐36 741 enhances osteogenic differentiation and reduces osteoporosis following ovariectomy by modulating the m6A methylation of BMP2 transcripts through METTL3. In essence, the comprehensive elaboration on the crosstalk between major methylation modifications and piRNA could provide clear clues for their roles in facilitating cancer oncogenesis. Nevertheless, the functions of m6A modifications in UC remain unclear and the precise mechanism by which PIWI protein directs RNA methylation is inadequately understood.

#### 1.2.2 piRNA Mediated Transposable Elements Silencing

PiRNA are widely recognized as pivotal participants in the silencing mechanism of transposable element [14, 16, 17, 18, 20](TE). TE, a functional DNA fragment, can continuously move its position within the genome of the organism with the same or different copies. Additionally, TE potentially contributes to the induction of genomic instability in the process of tumor development [56, 57, 58, 59]. Mechanistically, two distinct modalities have been reported underlying the mechanistic intricacies [60]. Firstly, piRNA production targeting evolutionarily younger TEs occurs independently of piRNA clusters. Secondly, piRNAs that target older TEs work in concert with cluster‐independent piRNA production to achieve broad targeting of nearly all TEs in the postnatal testis [60].

The germ‐cell‐specific expression of HENMT1 and PIWI proteins is indispensable for the regulation of transposons, underscoring their pivotal roles in transposon control within the germ cell lineage [61]. Liu et al. [62] found that the gene HENMT, which is crucial for piRNA biogenesis, particularly within the Ping‐Pong cycle, was downregulated in the large genome grasshopper, suggesting its role in the regulation of transposable elements. This reduction alters the previously established beneficial relationship between TEs and piRNA levels. Yuan et al. [63] present a computational approach to identify piRNA targets on mouse mRNAs using a support vector machine classifier trained with Miwi CLIP‐Seq and position‐derived features. The results suggest that piRNAs may regulate a significant number of protein‐coding genes, with predicted targets showing expression changes when Miwi slicer activity is disrupted. This study highlights the potential broad role of piRNAs in gene expression regulation beyond TE silencing, particularly during spermatogenesis. Notably, such methodologies exhibit limitations in identifying insertions of uncharacterized TEs and fail to assemble complete sequences of inserted elements. In addition, Ellison et al. [64] employed advanced methodologies, specifically leveraging nanopore sequencing and Hi‐C scaffolding techniques, to systematically generate denovo genome assemblies. The principal role ascribed to piRNA is the suppression of TE activities, where TEs represent repetitive DNA sequences capable of mobilizing across the genome. The ongoing challenge within the genomics domain involves elucidating the nuanced details of how piRNA intricately integrate and organize TEs, underscoring the complexity inherent in this regulatory process.

### 1.3 Databases of piRNAs

Several bioinformatics resources have been developed to facilitate the investigation of piRNAs. A pivotal contribution to this domain would be the establishment of a piRNA database, created for the comprehensive gathering, cataloging, and architectural arrangement of piRNAs (Table 2). Notable platforms include piRBase [65, 66, 67], piRNAQuest [68] and piRNA‐eQTL [69]. To address the intersection of piRNA and disease, the piRDisease was established by Xin et al. [70], encapsulating 7939 manually curated associations that encompass 4796 experimentally supported piRNAs implicated in 28 diseases. These resources collectively serve as invaluable tools, empowering researchers to advance their understanding of unresolved queries surrounding piRNA biogenesis and their nuanced modes of action.

**TABLE 2:** The information of piRNA database.

Database	Website	Characteristic	References
piRBase	http://bigdata.ibp.ac.cn/piRBase/	The largest collection of piRNA among existed databases and contains 77 million piRNA sequences from 9 organisms	[66]
piRBase version 3.0	http://bigdata.ibp.ac.cn/piRBase/	Including the comprehensive annotation of piRNA sequences, the increasing number of piRNA, the potential information of piRNA targets and disease related piRNA	[67]
piRNAQuest	http://bicresources.jcbose.ac.in/zhumur/pirnaquest/	The comprehensive database of 41 749 human, 890 078 mouse and 66 758 rat piRNA obtained from National Center for Biotechnology Information (NCBI) and different small RNA sequence experiments	[68]
piRNA‐eQTL	http://njmu‐edu.cn:3838/piRNA‐eQTL/	The gathered genotyping and piRNA expression data from 10 997 samples spanning 33 cancer types from The Cancer Genome Atlas (TCGA)	[69]
piRDisease	http://www.piwirna2disease.org/index.php	Offer detailed information of the piRNA in respective disease, such as experimental support, brief description, sequence, and location information	[70]
PingPongPro	https://github.com/suhrig/pingpongpro	The scrutiny of small RNA‐Seq data to discern indicators of ping‐pong cycle activity and facilitating accurate identification of transposable elements subject to suppression through the ping‐pong cycle	[71]
piRTarBase	http://cosbi6.ee.ncku.edu.tw/piRTarBase	Predicted and experimentally identified piRNA targeting sites in *Caenorhabditis elegans*	[72]
piRNAclusterDB	http://www.smallrnagroupmainz.de/piRNAclusterDB.html	Provide comprehensive data on piRNAs clusters in multiple species, tissues and developmental stages based on small RNA sequence data deposited at NCBI's Sequence Read Archive (SRA)	[73]

The mRNA fragments generated initiate a positive feedback mechanism known as the ping‐pong cycle, which bolsters piRNA synthesis and intensifies the silencing effect on transposons. Due to this function, the computational tool PingPongPro has been developed under the GPLv3 license [71]. Recent investigations have elucidated the potential of piRNA to silence diverse endogenous genes, prompting Wu et al. [72] to develop piRTarBase. Bioinformatics pipelines for analyzing piRNAs predominantly concentrate on piRNAs that originate from the germline and the formation of piRNA clusters. Over the past few years, both the quantity of small RNA sequencing data produced and the variety of species subjected to such analysis have expanded swiftly. Despite the proliferation of piRNA sequence data, Rosenkranz et al. [73] note the scarcity of understanding regarding the regulatory factors influencing piRNA clusters and the evolutionary shifts responsible for their emergence or disappearance. To tackle these topics, Rosenkranz [73] has created an accessible piRNA cluster database. Those online databases provide sources about the sequence of piRNA, potential target genes, and target TE, serving as valuable resources for researchers. However, compared with miRNA [74, 75], available bioinformatics resources for piRNA are limited to a large extent.

### 1.4 PiRNA Detection Technology and Clinical Application

#### 1.4.1 Detect piRNA of Urologic Carcinoma in Extracellular Vesicles

**FIGURE 5:**
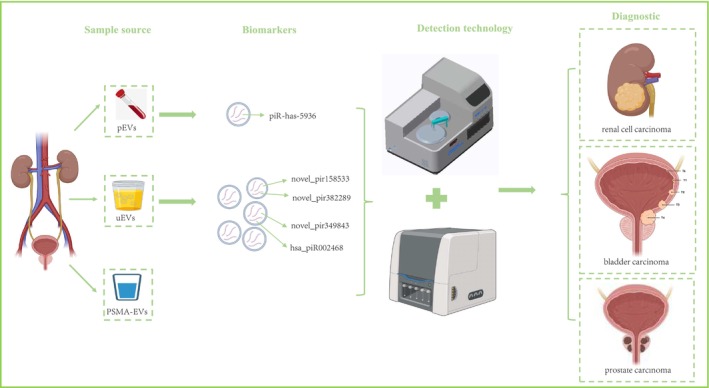
A non‐invasive diagnostic approach based on extracellular vesicles of Urologic Carcinoma: plasma extracellular vesicles (pEVs), urinary extracellular vesicles (uEVs), prostate‐specific membrane antigen extracellular vesicles (PSMA‐eEVs).

Several technological advancements have been introduced for the detection of piRNAs within extracellular vesicles (EVs). Bajo‐Santos et al. [76] performed RNA sequencing analysis on plasma extracellular vesicles (pEVs) and urinary extracellular vesicles (uEVs) collected pre‐radical prostatectomy and post‐radical prostatectomy. Assessing the diagnostic value of piRNA derived from uEVs for PC, the expressions of novel_pir349843, novel_pir382289, novel_pir158533, and hsa_piR002468 in uEVs were notably higher in individuals with pancreatic cancer, indicating their potential as biomarkers for non‐invasive PC diagnosis (Figure 5) [77]. Saboetal. [27] utilized next‐generation sequencing; the small non‐coding RNAs within pEVs were profiled, revealing decreased levels of miR‐185‐5p and miR‐106a‐5p, or increased levels of miR‐10b‐5p in these vesicles, which were found to be related to worse survival and exhibited diagnostic potential in BC, especially in advanced grade (Figure 5).

In conclusion, EVs represent a promising low‐invasive source of biomarkers with the potential for diagnosing, monitoring prognosis, or evaluating treatment efficacy. However, the sensitivity and specially is not enough for utilizing in clinic for UC. The application of new and efficient technologies or the combination with other biomarkers holds promise in improving sensitivity and specificity for clinical applications.

#### 1.4.2 Transform Technology for piRNA and piRNA Cluster in Cancer

Continuous advancements in high‐throughput sequencing technologies and bioinformatics analyses are poised to facilitate more comprehensive and accurate profiling of piRNAs across various cancer types. Improvements in the detection of piRNAs from mammalian cells have been achieved by cross‐linking RNA to nylon membranes [78]. The process likely improves the quality and/or positioning of RNA on the membrane, reducing degradation and enhancing the signal detection [78]. It is compatible with various types of hybridization probes and offers a simple, cost‐effective alternative to UV cross‐linking [78]. In an earlier investigation of microRNAs in shark liver, several piRNAs were identified among the small RNAs sequenced using Solexa technology. In a pioneering study, Yang et al. [79] employed Solexa sequencing to detect piRNAs in the liver of the whitespotted bamboo shark and also shed light on the regulatory impact of these piRNAs within the liver. Due to producing longer transcripts with higher read coverage, NorahDesk is suitable to predict both known and novel piRNAs [80]. Additionally, a novel method involving stem‐loop primer reverse transcription followed by quantitative qRT‐PCR was established to quantify piRNA‐54 265 levels [81]. To clarify this important issue, Mai et al. [81] carried out supplementary experiments to confirm the validity of piRNA detection protocols and to authenticate the presence of piR‐54 265 in samples of human serum. In addition, for clinical laboratory testing easily, Kawakami et al. [82] devised a novel sandwich ELISA technique for the detection of prostate‐specific membrane antigen(PSMA) on EVs, utilizing T‐cell immunoglobulin domain and mucin domain‐containing protein 4 (Tim4) as an EV capture molecule (Figure 5). This can offer diagnostic potential in UC and enhance the assay's sensitivity and specificity (Figure 5). This technology makes clinical detection of piRNAs possible, but not effective for piRNAs clusters.

Currently, the identification of piRNA clusters is predominantly reliant on bioinformatics approaches, rather than the rigor of the detection methods. Despite the growing amount of piRNA sequence data available, there is a noted scarcity in computational studies dedicated to the detection of piRNA clusters, and there is a recognized need for improvement in both the effectiveness and efficiency of the tools designed for piRNA cluster detection. To solve this problem, Rosenkranz et al. [83] developed a software tool known as probabilistic TRacking and Analysis of Clusters (proTRAC), a developed software, to identify piRNA clusters from piRNA sequences in the respective species including human, macaque, mouse, and rat. Subsequently, the findings were validated against the piRNA cluster annotations provided by piRNABank [84]. This analysis revealed discrepancies between proTRAC‐identified clusters and those annotated in piRNABank, emphasizing the need for refined methodologies. On the other hand, for piRNA detection, Liu et al. [85] developed a support vector machine‐based method, incorporating features including k‐mer weighting, k‐mer with wildcards, position‐specific base preferences, and piRNA length considerations. This approach exhibited favorable precision and sensitivity, approximately reaching 90%. To encapsulate, computational research has the potential to boost the precision of piRNA cluster detection as well as the precision of identifying individual piRNAs.

## 2 Conclusion and Perspective

The dysregulation of piRNA has been implicated in influencing TEs, m6A methylation, and other molecular processes, thereby contributing to the progression and prognosis of cancer [86, 87, 88, 89, 90]. Altered expression patterns of specific piRNA have shown associations with distinct clinical outcomes in cancer patients [91, 92, 93, 94], suggesting a potential role for piRNA as prognostic indicators capable of predicting disease progression and guiding treatment decisions. Furthermore, changes in piRNA expression profiles observed during treatment may serve as indicative markers of treatment response or resistance. Consequently, monitoring piRNA holds the promise of furnishing valuable information for optimizing treatment strategies.

The identification and characterization of specific piRNA or related pathways implicated in UC are areas of active investigation [24]. While certain studies have underscored the crucial roles played by specific piRNA in UC, the intricate mechanisms underlying these regulatory processes remain incompletely understood [48]. Additionally, challenges persist in elucidating detailed mechanisms, and further breakthroughs are required to comprehensively unravel the complexities associated with piRNA‐mediated regulation in UC. Firstly, a significant challenge within the realm of piRNA research is the lack of standardized nomenclature, which can impede consistent communication and data analysis across studies [94]. Various identifiers such as DQ594040, has_piR_002468, piR‐51 810, among others, are employed for denoting piRNA, resulting in a lack of uniformity in nomenclature. Addressing this issue is imperative for enhancing clarity and consistency in piRNA‐related studies. Secondly, despite promising preclinical research outcomes, the translation of piRNA‐related findings into clinical applications, particularly therapeutic interventions, is still nascent. The transition from preclinical promise to robust clinical applications necessitates further exploration, optimization, and validation. The third challenge pertains to the limited understanding of the long‐term effects and potential side effects associated with manipulating piRNAs in a clinical setting. The intricate regulatory roles of piRNA in various cellular processes underscore the importance of comprehensive investigations to delineate the consequences and potential risks of therapeutic piRNA manipulation.

While preclinical research offers encouraging prospects, the translation of piRNA‐based approaches to the clinical realm requires meticulous consideration of safety, efficacy, and standardized nomenclature. Combining piRNA‐based strategies with existing treatments, such as siRNAs, cell‐based microrobots [95], and gene editing technologies, holds promise for enhancing therapeutic outcomes and overcoming resistance mechanisms. For instance, nanotherapeutic systems containing piR‐1742 inhibitors have the potential to significantly inhibit the growth and spread of RCC [36]. Furthermore, cell‐based microrobots are engineered by transforming natural cells to possess characteristics of robots [96]. These microrobots integrate the biological activities of cells with materials, enabling them to harness various actuation methods, achieving autonomous navigation and sensing for precise therapeutic interventions [97]. The OA‐loaded cell‐based microrobots with virotherapy can improve anticancer efficacy for BC patients [95].

The continuous evolution of RNA‐based therapeutics may provide valuable tools for refining piRNA‐targeted interventions in cancer [98, 99, 100, 101, 102]. For the drug‐resistant patients, combined treatment of piRNA‐targeted interventions and carcinoma inhibitors, such as circPTEN [103], may improve therapeutic effectiveness. As these endeavors progress, comprehensive and long‐term studies become indispensable for evaluating the safety and efficacy of any piRNA‐based therapies. Positive outcomes from preclinical and subsequent clinical trials may pave the way for regulatory approval and clinical integration, thereby offering novel therapeutic avenues for cancer patients. In future clinical applications, piRNA exhibits several key aspects. PiRNA and PIWI proteins are aberrantly expressed in urologic carcinoma, and these molecules possess high stability, potentially serving as diagnostic and prognostic biomarkers as well as therapeutic targets for cancer. A thorough discussion based on the biogenesis of piRNA elucidates its role and underlying mechanisms in various diseases, which can further be utilized to develop piRNA‐related patents for prevention, diagnosis, and treatment [104]. Thereby enriching the molecular biology theory of piRNA and providing a theoretical foundation for its clinical application. However, the current lack of highly sensitive detection technologies for piRNA hinders its rapid translation into clinical practice.

## Author Contributions

J.C., H.F., and S.W. developed the core research idea and implemented the systematic retrieval strategy for data collection (e.g., database selection, keyword optimization). J.C. and S.J. authored the main body of the review text. J.C. created and annotated all figures to visually represent key concepts and results. D.L. and G.M. designed and formatted all tables to systematically present datasets and comparisons. M.W., Y.L., D.S., and Y.L. contributed to revising the manuscript for technical accuracy and clarity. J.C., S.J., H.F., and Y.L. focused on technical accuracy, verifying claims against cited evidence and correcting logical inconsistencies. All authors offered valuable suggestions for improving the overall quality and presentation of this paper.

## Disclosure

The figures are reproduced with permission/published under an open license and these licenses permit reuse and adaptation.

## Conflicts of Interest

The authors declare no conflicts of interest.

## Data Availability

Data supporting the figures and tables of this study are available from the corresponding authors upon reasonable request.
